# Clinical indications and outcomes of Impella devices for severe cardiogenic shock in COVID-19 patients: a systematic review

**DOI:** 10.1097/MS9.0000000000001425

**Published:** 2023-11-01

**Authors:** Bahadar S. Srichawla, Vincent Kipkorir, Manraj Sekhon

**Affiliations:** a Department of Neurology, University of Massachusetts Chan Medical School, Worcester, Massachusetts; b Department of Internal Medicine, University of California Riverside School of Medicine, Riverside, California, USA; c Department of Medicine, University of Nairobi, Nairobi, Kenya

**Keywords:** BiPella, cardiogenic shock, COVID-19, ECMO, extracorporeal life support, fulminant myocarditis, Impella, SARS-CoV-2

## Abstract

**Background:**

Coronavirus disease 2019 (COVID-19) can present with significant cardiac dysfunction, including cardiogenic shock. Mechanical circulatory support with an Impella device may be utilized in these patients to support and offload native right ventricle (RV) and left ventricle (LV) functions. This systematic review aims to describe clinical indications, management, laboratory data, and outcomes in patients with severe cardiogenic shock from COVID-19 treated with an Impella device.

**Methods:**

A PRISMA-directed systematic review was performed and prospectively registered in PROSPERO. The databases accessed included PubMed/MEDLINE, Scopus, and ScienceDirect. Quality and risk of bias assessments were completed using the Joanna Briggs Institute (JBI) checklist for case reports.

**Results:**

A total of 16 records were included in the qualitative synthesis; 8/16 (50%) of the patients were men. The average age was 39 years (SD: 14.7). The biventricular Impella (BiPella) approach was recorded in 3/16 (18.75%) patients. A total of 4/16 (25%) individuals required renal replacement therapy (RRT). Single-device usage was observed in three cases: 2/16 Impella CP (12.5%) and 1/16 Impella RP (6.25%). Treatment of COVID-19 myocarditis included a wide range of antivirals and immunomodulators; 8/16 (50%) cases needed ECMO (extracorporeal membrane oxygenation) support. Overall, only 2/16 (11.7%) individuals died.

**Conclusions:**

Sixteen reported individuals have received an Impella implanted with a mortality rate of 11.7%. Concurrent use of RRT and ECMO implantation was often observed. Overall, the Impella device is an effective and safe strategy in the management of COVID-19-related cardiogenic shock. Future studies should include long-term results.

## Introduction

Highlights

- Coronavirus disease 2019 (COVID-19) can lead to significant cardiac dysfunction, including cardiogenic shock, necessitating mechanical circulatory support.
- The Impella device is used to support and offload the native right ventricle (RV) and left ventricle (LV) function in patients with severe cardiogenic shock from COVID-19.
- The study included 16 patients, with 50% being men and an average age of 39 years. Renal replacement therapy (RRT) was required in 25% of cases.
  The biventricular Impella (BiPella) approach was recorded in 3/16 (18.75%) individuals; 4/16 (25%) individuals required RRT. Single-device usage was observed in three cases: 2/16 Impella CP (12.5%) and 1/16 Impella RP (6.25%).
- The mortality rate among the reported cases was 11.7%. Concurrent use of RRT and extracorporeal membrane oxygenation (ECMO) support was observed. The Impella device was deemed effective and safe for managing COVID-19-related cardiogenic shock.

The coronavirus disease 2019 (COVID-19) pandemic has resulted in a sudden and significant increase in the number of patients with cardiac disease. As a result, there has been growing interest in the use of advanced medical technologies, such as the Impella device, to assist with the management of severe cardiac dysfunction in these patients. COVID-19 can cause severe respiratory and cardiac complications, including cardiogenic shock. Studies have shown that patients with COVID-19 who develop cardiogenic shock have a mortality rate of more than 50%^1^. The Impella device (ABIOMED, Danvers, Massachusetts, USA) is a percutaneous left or right ventricular assist device that provides hemodynamic support throughout the entire cardiac cycle^2^.

Clinically, the Impella device is commonly indicated for use in patients suffering from acute myocardial infarction complicated by cardiogenic shock, high-risk percutaneous coronary intervention, and other severe heart failure conditions. Hemodynamic indicators supporting Impella implantation include a cardiac power output (CPO) of less than 0.6 W and pulmonary artery pulsatility index (PAPi) of less than 0.9^3^. The approach to implantation usually involves percutaneous insertion via femoral artery access, guided by fluoroscopy. The Impella catheter is threaded through the aortic valve into the left ventricle (LV), where it helps to pump blood from the LV into the ascending aorta, thereby improving cardiac output and end-organ perfusion^4,5^.

This systematic review aims to provide a comprehensive synthesis of current evidence on the use of the Impella device in patients with COVID-19-mediated cardiac disease. The review will critically evaluate available studies on the clinical presentation, biomarkers, extracorporeal life support techniques, hemodynamic parameters, and outcomes of Impella use in this population of patients. The findings of this review will provide valuable information on the current state of evidence on Impella use in COVID-19-mediated cardiac disease and will inform clinical decision-making in this rapidly evolving field.

## Methods

This systematic review was completed in accordance with the Preferred Reporting Items for Systematic Reviews and Meta-Analyses (PRISMA) and Assessing the Methodological Quality of Systematic Reviews (AMSTAR) guidelines^6,7^. The AMSTAR 2 checklist was completed for this systematic review protocol and a high-level of compliance was determined. The review was registered with The International Prospective Register of Systematic Reviews (PROSPERO) and at researchregistry.com.

### Data sources and search strategy

The purpose of this systematic review is to identify cases of COVID-19 complicated by cardiogenic shock requiring single or biventricular Impella (BiPella) support for management. A systematic review of the literature was conducted using PubMed/PubMed Central/MEDLINE, Scopus, and ScienceDirect databases. When able, a combination of medical subject headings (MeSH) terms was used to incorporate database indexing. Boolean operators (OR and AND) were used to create a comprehensive search strategy. The following search string was utilized: ((“Impella”[MeSH Terms] OR “Impella” [Title/Abstract]) AND (“Cardiogenic Shock”[MeSH Terms] OR “Heart Failure”[MeSH Terms])) AND (“COVID-19”[MeSH Terms] OR “Coronavirus”[MeSH Terms] OR “SARS-CoV-2”[MeSH Terms]). A gray literature search was completed by searching the first 10 pages of GoogleScholar to include relevant articles. The review search was completed on 10 March 2023. Only peer-reviewed journal articles were included. Backward and forward citation was utilized when appropriate to incorporate more articles in the review.

### Data characterization

Studies will be included if they meet the following criteria: (1) Records published from 2019 to the present. (2) Reporting on the use of Impella for cardiogenic shock secondary to COVID-19. (3) Reporting on efficacy, biomarkers, clinical presentation, hemodynamic parameters, and outcomes of Impella use in COVID-19 patients. (4) Published in the English, Punjabi, Hindi, and Urdu languages (native to the authors). All included records are confirmed to have SARS-CoV-2 (severe acute respiratory syndrome coronavirus 2) infection assessed via polymerase chain reaction (PCR). Articles that passed the screening and inclusion criteria were considered for analysis. Data extraction was conducted by two independent reviewers (B.S.S. and M.S.). For each study, the following information was extracted: name of the author, year of publication, age, gender, relevant laboratory findings including serum troponin, initial hemodynamic parameters, mechanical circulatory support device(s) used, use of inotropes, vasopressors, and outcomes.

### Data extraction and analysis

The data obtained were stored in Microsoft Excel (Microsoft Corporation, 2022) for cleaning, validation, and coding. Descriptive statistics were used to summarize the data. Frequencies and percentages were utilized to describe nominal data. Data were presented in a tabular manner when appropriate. Data analysis and visualization were completed on GraphPad Prism 8.4.3 for Windows, GraphPad Software, San Diego, California, USA, and Python 3.1.0 (64-bit) programming language (Python Software Foundation).

### Quality and risk of bias assessment

To minimize the risk of bias and assess our study’s methodological quality, the validated Joanna Briggs Institute (JBI) tool for case reports/series was utilized. The risk of bias was assessed independently by two authors (B.S.S. and M.S.). Additionally, a third reviewer was available to resolve discrepancies between reviewers. The protocol was developed and registered on PROSPERO before beginning the systematic review.

## Results

### Search results

**Figure 1:**
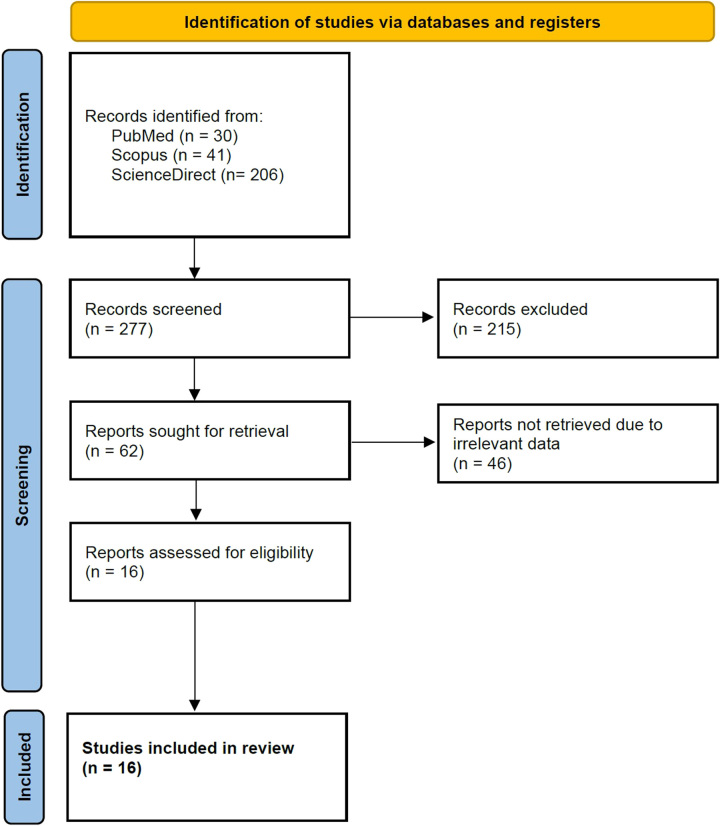
PRISMA flow diagram for search protocol. PRISMA, Preferred Reporting Items for Systemic Reviews and Meta-Analyses.

A total of 277 records were screened; 215 records were excluded because they were not published in peer-reviewed academic journals and/or case reports/series on the topic, 62 reports were sought for retrieval, and 46 were excluded due to irrelevant data. Sixteen records were screened for eligibility and included in the analysis^8–23^. A flow diagram is included showing the literature search results (Fig. 1).

A total of 8/16 (50%) of the patients were men. The average age was 39 years (SD: 14.7). The BiPella approach was observed in 3/16 (18.75%) of the cases with favorable outcomes, including discharge from the hospital within 30 days and limited morbidity after discharge^8,20,21^. Furthermore, the report by Srichawla and Sekhon^21^ describes the first reported use of the BiPella approach in a pregnant patient. Single-device usage was seen in three cases: 2/16 (12.5%) Impella CP and 1/16 (6.25%) Impella RP. A total of 4/16 (25%) individuals required renal replacement therapy (RRT), which includes continuous veno-venous hemofiltration/hemodialysis (CVVH/HD) and intermittent hemodialysis (iHD). 8/16 (50%) and 9/16 (56.25%) patients required inotropes or vasopressors, respectively. Treatment of COVID-19 myocarditis included a wide range of antivirals and immunomodulators, including intravenous methylprednisolone (IVMP), intravenous immunoglobulin (IVIG), remdesivir, anakinra, and convalescent plasma. The case of Cohen *et al*.^13^ was complicated by a pulmonary embolus and required right ventricle (RV) support with the Impella RP device and anticoagulation. The case by Kaki *et al*.^15^ involved thrombectomy of a right atrial thrombus in addition to Impella CP and extracorporeal membrane oxygenation (ECMO). 8/16 (50%) of the cases needed ECMO, including veno-arterial (VA), veno-venous (VV), and veno-veno-arterial (VVA). Common initial biomarkers reported included an elevated C-reactive protein (CRP), white blood cell (WBC) count, troponin, and pro-NT BNP. Overall, only 2/16 (11.7%) individuals died after receiving mechanical circulatory support for COVID-19-mediated cardiogenic shock. Table 1 provides a summary of the search results.

**Table 1:** Identified cases of Impella support in COVID-19-related cardiogenic shock

Reference	Age	Gender	Initial serum lab findings	Initial troponin (ng/ml)	Peak troponin (ng/ml)	Initial hemodynamic measurements	Extracorporeal life support (ECLS)	Renal replacement therapy	Inotropes	Vasopressors	Medical management	Outcomes
Ajello *et al*. (2022)^8^	49	M	WBC: 23.7, CRP: 490 mg/dl, creatinine: 4.38 mg/dl, D-dimer: >20 mcg/ml	280	28	PCWP: 19 mmHg, CVP 19 mmHg, CI 2.06 l/min/m^2^, PAP: 38/21/26	Impella RP + Impella CP (BiPella)	No	No	No	BiPella explanted on day 10.	Discharge within 30 days. No heart failure at 6 months.
Apostolidou *et al*. (2021)^9^	7	F	Leukocytosis, elevated CRP (unspecified)	—	—	FS 10%	Impella CP	Yes	Yes	No	Monoclonal antibodies, dexamethasone, remdesivir, anakinra.	Death on day 37 of hospitalization.
Asakura *et al*. (2022)^10^	49	M	—	—	—	LVEF: 30%	Impella CP (5.0)	No	Yes	Yes	Methylprednisolone	Discharged on day 39 of hospitalization with no signs of heart failure.
Bemtgen *et al*. (2021)^11^	18	M	CRP: 105.9 mg/dl, IL-6: 128 pg/ml, PCT: 0.12 ng/ml	—	341	CO: 3.6 l/min, PCWP: 26 mmHg	Impella CP + VA ECMO	Yes	No	No	Dexamethasone, anakinra, IVIG.	MCS was discontinued on day 7. The patient was discharged on day 32.
Bemtgen *et al*. (2020)^12^	52	M	CRP: 6.4 mg/dl, PCT: 0.22 ng/ml, proBNP: 2274 pg/ml, WBC: 6.03	—	—	CI: 1.8 l/min/m^2^	Impella CP (day 3—19) and VA + ECMO (day 4—11) VV ECMO (day 11—22)	No	Yes	Yes	Lopinavir, ritonavir, chloroquine, levosimendan.	MCS was discontinued on day 22.
Cohen *et al*. (2022)^13^	57	F	—	—	—	—	Impella RP	No	No	No	Anticoagulation for PE	One-year follow-up showed normal RV systolic function via ECHO.
Ebert *et al*. (2022)^14^	33	F	NT-proBNP: 4791 pg/ml (peak)	—	—	LVEF: 19%	Impella CP + VVA-ECMO	No	No	Yes	—	Impella CP was explanted on day 5, VVA-ECMO was removed on day 9.
Kaki *et al*. (2020)^15^	57	F	WBC: 27.35, creatine: 2.2 mg/dl, lactic acid: 6.8 mmol/l, CRP: 266.9 mg/dl, ferritin: 3233 ng/ml	0.21	—	SBP: 50 mmHg, RAP: 20 mmHg, MVO_2_: 42%	RP Impella + ECMO	Yes	No	No	Thrombectomy (RA thrombus), vasopressors.	Discharge on day 23.
Mahrokhian *et al*. (2021)^16^	65	M	Creatinine: 3.0 mg/dl	—	—	LVEF <10%; MAP ~60	CP Impella + VA ECMO	No	Yes	Yes	Anti-arrhythmic, remdesivir, dexamethasone.	Removal of Impella on day 21. LVEF of 40% on day 23.
Newman *et al*. (2022)^17^	49	M	WBC: 19.6, lactic acid: 5.1 mmol/l, creatinine: 3.0 mg/dl, CRP: 39.3 mg/dl, D-dimer: 3.62 μg/ml	—	—	MAP: 65 mmHg, RAP: 15 mmHg, PAP: 32/20 mmHg, PCWP: 21 mmHg, PAS: 27.4%, CO: 3.7 l/min, CI: 1.9 l/min/m_2_, SVR: 10 81l dynes/s/cm^5^	CP Impella + VA ECMO	No	No	Yes	IVMP, IVIG.	VA-ECMO was discontinued on day 5. Impella was removed on day 6. Discharged on day 11.
Noone *et al*. (2022)^18^	38	F	WBC: 12.5, CRP: 127 mg/dl, IL-6: 300 pg/ml	140 pg/ml	450 pg/ml	LVEF 20%	Impella CP + VA ECMO	No	Yes	Yes	12.5 mg of levosimendan, ivabradine 5 mg, RegenCOV.	VA ECMO was explanted on day 7. Impella CP was explanted on day 8. Discharge on day 15.
Papageorgiou *et al*. (2022)^19^	43	M	NT-proBNP: 6100 ng/l, PCT: 0.06 mcg/l, lactate: 3.4 mmol/l, ferritin: 220 mcg/l	590	—	CO: 3.8 l/min, SBP: 85 mmHg, PP: 15 mmHg, LVEF: 10—15%, ScvO_2_: 53%	Impella CP + VA ECMO	No	Yes	Yes	Cefotaxime, colchicine, hydrocortisone.	MCS was discontinued on day 7.
Ruiz *et al*. (2021)^20^	35	F	NT-proBNP: 7139 pg/ml	0.28	—	EF <10%, RAP 21 mmHg, PAP 26 mmHg, PCWP 18 mmHg, CO: 2.1 l/min, CI 1.2 l/min/m^2^	Impella CP + Impella RP (BiPella)	No	No	Yes	—	MCS was discontinued on day 14. Discharge on day 23 with LVEF 60%.
Srichawla and Sekhon (2023)^21^	29	F	Lactic acid: 2.2 mmol/l, CRP: 1.1 mg/dl	1.45	70	PAPi: 0.3, CVP: 16.5 mmHg, CPO: 0.6 W	Impella RP + Impella CP (BiPella)	Yes	Yes	Yes	Remdesivir, dexamethasone.	BiPella explanted on day 17. Full recovery at 1-year follow-up.
Valchanov *et al*. (2020)^22^	43	M	WBC: 25.9, ferritin: 1430.9 mcg/l, CRP: 167 mg/l, D-dimer: 2296 ng/l, IL-6: 511 pg/ml, TNF-α: 13.89 pg/ml	509	—	LVEF <5% (day 3 before Impella placement)	VA-ECMO (day 1) + Impella CP (day 3)	No	Yes	Yes	Piperacillin-tazobactam, azithromycin.	Death on day 21 of hospitalization.
Yeleti *et al*. (2021)^23^	25	F	CRP: 3.0 mg/dl, BNP: 117 pg/ml, IL-6: 16.5 pg/ml, ferritin: 935 ng/ml, creatinine: 1.05 mg/dl, ALT: 34 U/l	10.65	—	Biventricular failure LVEF 5—10%	Impella RP + Impella CP. Transition to VA-ECMO + Impella CP	No	No	No	IVMP, Remdesivir, convalescent plasma.	Preserved LVEF at 4-month follow-up.

BiPella, biventricular Impella; CI, cardiac index; CO, cardiac output; CP Impella, cardiac power left ventricular Impella; CPO, cardiac power output; CRP, C-reactive protein; ECHO, echocardiogram; FS, fractional shortening; IL-6, interleukin-6; IVIG, intravenous immunoglobulin; IVMP, intravenous methylprednisolone; LDH, lactate dehydrogenase; LV, left ventricle; LVEF, left ventricular ejection fraction; MAP, mean arterial pressure; MCS, mechanical circulatory support; NT-ProBNP, N-terminal pro-brain natriuretic peptide; PAP, pulmonary artery pressure; PAPi, pulmonary artery pulsatility index; PAS, pulmonary artery saturation; PCT, procalcitonin; PCWP, pulmonary capillary wedge pressure; PE, pulmonary embolism; ProBNP, pro brain natriuretic peptide; RP Impella, right ventricular Impella; RRT, renal replacement therapy; RV, right ventricle; ScvO_2_, central venous oxygenation; SVR, systemic vascular resistance; TNF-α, tumor necrosis factor-alpha; VA-ECMO, veno-arterial extracorporeal membrane oxygenation; WBC, white blood cell.

### Quality and risk of bias assessment

The JBI critical appraisal and risk of bias were completed for all 16 records included within this study; 3/16 articles scored 7/8 points, consistent with a minimal risk of bias. Table 2 provides a complete checklist for all included records.

**Table 2:** Joanna Briggs Institute critical appraisal and risk of bias results for included records

Reference	Q1	Q2	Q3	Q4	Q5	Q6	Q7	Q8	Overall	Risk
Ajello *et al*. (2022)^8^	Y	Y	Y	Y	Y	Y	Y	Y	8	Low
Apostolidou *et al*. (2021)^9^	Y	Y	Y	Y	Y	N	Y	Y	7	Low
Asakura *et al*. (2022)^10^	Y	Y	Y	Y	Y	Y	Y	Y	8	Low
Bemtgen *et al*. (2021)^11^	Y	Y	Y	Y	Y	Y	Y	Y	8	Low
Bemtgen *et al*. (2020)^12^	Y	Y	Y	Y	Y	Y	Y	Y	8	Low
Cohen *et al*. (2022)^13^	Y	Y	Y	Y	Y	N	Y	Y	7	Low
Ebert *et al*. (2022)^14^	Y	Y	Y	Y	Y	Y	Y	Y	8	Low
Kaki *et al*. (2020)^15^	Y	Y	Y	Y	Y	Y	Y	Y	8	Low
Mahrokhian *et al*. (2021)^16^	Y	Y	Y	Y	N	Y	Y	Y	7	Low
Newman *et al*. (2022)^17^	Y	Y	Y	Y	Y	Y	Y	Y	8	Low
Noone *et al*. (2022)^18^	Y	Y	Y	Y	Y	Y	Y	Y	8	Low
Papageorgiou *et al*. (2022)^19^	Y	Y	Y	Y	Y	Y	Y	Y	8	Low
Ruiz *et al*. (2021)^20^	Y	Y	Y	Y	Y	Y	Y	Y	8	Low
Srichawla *et al*. (2023)^21^	Y	Y	Y	Y	Y	Y	Y	Y	8	Low
Valchanov *et al*. (2020)^22^	Y	Y	Y	Y	Y	Y	Y	Y	8	Low
Yeleti *et al*. (2021)^23^	Y	Y	Y	Y	Y	Y	Y	Y	8	Low

Q1: Were the patient’s demographic characteristics clearly described? Q2: Was the patient’s history clearly described and presented as a timeline? Q3: Was the current clinical condition of the patient on presentation clearly described? Q4: Were diagnostic tests or assessment methods and the results clearly described? Q5: Was the intervention(s) or treatment procedure(s) clearly described? Q6: Was the post-intervention clinical condition clearly described? Q7: Were adverse events (harms) or unanticipated events identified and described? Q8: Does the case report provide takeaway lessons? Overall: Sum of points.

Y – yes; N – no; U – unclear; NA –-not applicable.

## Discussion

This systematic review summarized 16 reported cases of Impella utilization in COVID-19-related cardiogenic shock. The pathophysiology of cardiogenic shock in patients with COVID-19 is not fully understood, but several mechanisms have been proposed. SARS-CoV-2 can infect cardiomyocytes and endothelial cells, leading to myocardial injury, inflammation, and endothelial dysfunction^24^. This can result in impaired cardiac function and the development of cardiogenic shock. Another potential mechanism is the systemic inflammatory response to SARS-CoV-2 infection. COVID-19 can trigger a cytokine storm, leading to widespread inflammation and endothelial damage. This can result in microvascular thrombosis and impaired tissue perfusion, leading to end-organ dysfunction and the development of cardiogenic shock^25^. Furthermore, hypoxemia and respiratory failure associated with severe COVID-19 can lead to increased pulmonary vascular resistance and right heart strain^26^. The case of Kaki *et al*.^15^ reported percutaneous RV thrombus aspiration complicated by acute RV failure requiring Impella RP implantation. Overall, the pathophysiology of cardiogenic shock in patients with COVID-19 is likely multifactorial, involving direct effects of the virus on the cardiovascular system, systemic inflammation and endothelial dysfunction, and lung complications^27^.

Despite advances in therapy, mortality from cardiogenic shock is ~40–60%^28^. Some quaternary care centers have developed a shock team to assess for rapid mechanical circulatory support. In one such institution, an increase in the survival rate was reported from 47% in 2016 to 57.9% in 2017 and 81.3% in 2018^29^. The National Cardiogenic Shock Initiative (NCSI) has identified multiple hemodynamic parameters indicated for mechanical circulatory support of the right and left ventricles^30^. A PAPi of 0.9 or less is associated with 100% sensitivity and 98% specificity to predict hospital mortality and is an indication for RV support^31^. Similarly, elevated lactate and decreased CPO are indicative of increased mortality and likely require LV support^3^. A CPO of less than 0.53 is associated with a mortality rate of ~50%^32^. Our review also showed that the Impella device can be successfully placed in the prone position^22^.

**Figure 2:**
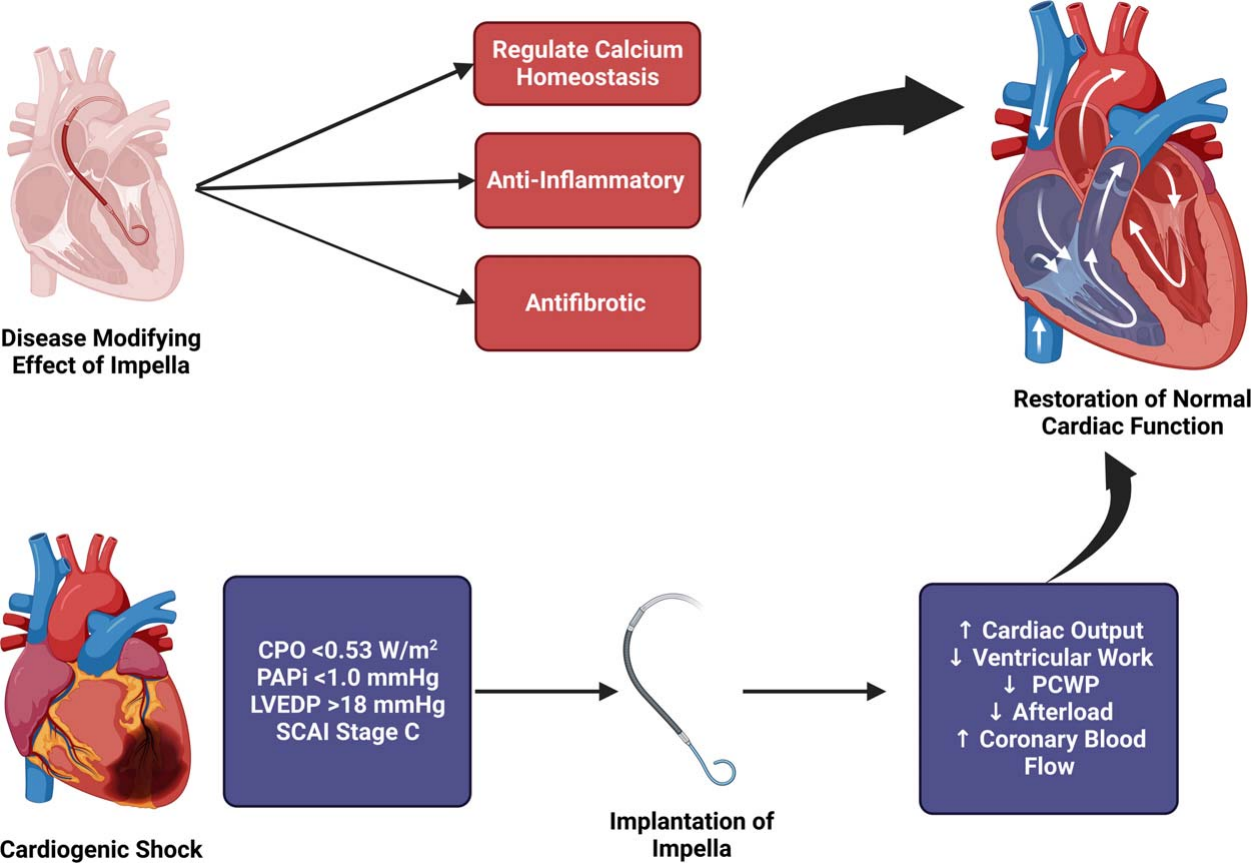
Primary and secondary disease-modifying effects of the Impella device in fulminant myocarditis and cardiogenic shock. CPO, cardiac power output; PAPi, pulmonary artery pulsatility index; LVEDP, left-ventricular diastolic end pressure; PCWP, pulmonary capillary wedge pressure; SCAI, The Society for Cardiovascular Angiography and Interventions.

The relatively low mortality rate observed in this systematic review is likely due to young age and the reversibility of viral myocarditis. The mortality rate is reportedly much higher in older adults with ischemic cardiomyopathy. Increased LV pressure and volume are associated with altered metabolic substrate utilization, decreased mitochondrial function, and energy production^33^. With durable left ventricular assist devices, it has previously been established that LV unloading is cardioprotective by reversing adverse cardiac remodeling and through antifibrotic effects^34,35^. Beyond serving the immediate effect of mechanical circulatory support, the Impella device is believed to also function as a disease-modifying treatment in fulminant myocarditis. These changes include a decrease in the inflammatory response due to LV and/or RV unloading, calcium homeostasis, and a decrease in cardiac immune cells, leading to improved myocardial recovery/remission^36^. Previous cohort studies have also shown that the Impella approach is more cost-effective than the conventional management of fulminant myocarditis^37^. Figure 2 provides an overview of the primary and secondary disease-modifying effects.

### Strengths, limitations, and future directions

The strengths of this systematic review include the use of a PRISMA guideline-directed approach and the prospectively registered protocol on PROSPERO, which can help ensure a comprehensive and unbiased search of the literature. The review also included quality and risk of bias assessments using the JBI checklist for case reports, which can help assess the reliability and validity of the included studies. This study has some limitations, including the small sample size, limited geographic distribution of the included studies, and notably, the absence of randomized controlled trials (RCTs). While our systematic review has sought to provide comprehensive insights into the use of Impella devices in COVID-19 cardiomyopathy patients, the lack of RCTs presents a significant limitation. Thus, our findings should be interpreted with caution, and there is a strong need for future research involving RCTs to validate and possibly refine these conclusions. Future clinical trials should take into consideration confounders in cases of out of hospital cardiac arrest. Heterogeneity, among urban and rural centers, may provide a more comprehensive understanding of extracorporeal life support^38^. Another limitation is the lack of detailed reporting on important comorbidities such as hypertension, heart failure, and diabetes in the studies we reviewed. Understanding the status and management of these comorbid conditions could offer additional insights into patient outcomes and the efficacy of Impella devices. Specifically, it would be beneficial to know how well-controlled these conditions are, as this could have a significant impact on treatment effectiveness and overall prognosis. Future studies should aim to include these data for a more comprehensive understanding of the treatment landscape. The retrospective nature of the studies may have introduced bias and confounders that were not considered in the analysis. The low mortality rate reported in this review may be due to a positive reporting bias.

The findings of this study have immediate and impactful implications for healthcare professionals managing patients with COVID-19 cardiomyopathy. Our review indicates that the Impella device can be a viable option for patients with severe cardiogenic shock that is refractory to standard medical therapies. It can guide healthcare providers in making timely and evidence-based decisions on initiating mechanical circulatory support. Given the device’s cost and the need for specialized expertise, medical directors can use the results of this study to allocate resources more effectively, balancing the benefits against the financial and logistical implications. Future directions of research in this area may include larger studies with longer follow-up periods to evaluate the long-term outcomes of the use of Impella devices in patients with COVID-19 with severe cardiogenic shock. More research is needed to better understand optimal management strategies for COVID-19 myocarditis, which can involve a wide range of antivirals and immunomodulators. Finally, studies may focus on refining the indications for mechanical circulatory support devices, such as Impella, in patients with COVID-19 with severe cardiac dysfunction, to identify which patients are most likely to benefit from this approach.

## Conclusions

This systematic review evaluated the use of Impella devices for mechanical circulatory support in patients with severe cardiogenic shock from COVID-19. The study found that various clinical indications, management strategies, and laboratory data were utilized in treating these patients. The average age of the patients was relatively young at 39 years, and 50% of the patients were men. The use of RRT was required in a quarter of the cases, and ECMO support was required in half of the cases. The mortality rate was relatively low, at 11.7%. These findings suggest that Impella devices may be a useful option for managing severe cardiac dysfunction in patients with COVID-19 with cardiogenic shock. The Impella device may also act as a disease-modifying therapy, improving mitochondrial function, myocardial energetics, calcium homeostasis, and exerting anti-inflammatory and antifibrotic effects. However, more research is needed to further evaluate the efficacy of this approach, particularly in larger patient populations and with longer-term follow-ups.

## Ethical approval

This research was conducted in compliance with the Declaration of Helsinki.

## Consent

No patient records were accessed in this systematic review.

## Sources of funding

No internal or external funding was received for this manuscript.

## Author contribution

B.S.S.: conceptualization, data curation, formal analysis, methodology, project administration, resources, software, validation, visualization, and writing – original draft, review, and editing; V.K.: conceptualization and writing – original draft, review, and editing; M.S.: writing – original draft.

## Conflicts of interest disclosure

On behalf of all authors, the corresponding author states that there are no conflicts of interest.

## Research registration unique identifying number (UIN)

PROSPERO UIN: CRD42023353615 https://www.crd.york.ac.uk/prospero/display_record.php?RecordID=353615.

Research Registry UIN: reviewregistry1708

https://www.researchregistry.com/browse-the-registry#registryofsystematicreviewsmeta-analyses/registryofsystematicreviewsmeta-analysesdetails/65175af8584a2a0027da7178/.

## Guarantor

Bahadar S. Srichawla.

## Data availability statement

Data are available for review based on a reasonable request from the Editor-in-Chief.

## Provenance and peer review

Not commissioned, externally peer-reviewed.
